# Effects of Video-Guided Active Breaks on Motor Competence of Schoolchildren with Special Education Needs

**DOI:** 10.3390/children12070820

**Published:** 2025-06-21

**Authors:** Alejandra Robles-Campos, Daniel Reyes-Molina, Karen Kracht-Suazo, Igor Cigarroa, Jaime Cárcamo-Oyarzun, Nicolas Martinez-Lopez, Margarita Perez-Ruiz, Alberto Grao-Cruces, Jorge Mota, Alberto Ruiz-Ariza, Fernando Muñoz Hinrichsen, Guillermo García-Pérez-de-Sevilla, Carlos Celis-Morales, Rafael Zapata-Lamana

**Affiliations:** 1 Escuela de Educación, Universidad de Concepción, Los Ángeles 4451032, Chile; alejandrarobles@udec.cl (A.R.-C.); rzapatal@santotomas.cl (R.Z.-L.); 2 Escuela de Kinesiología, Facultad de Salud, Universidad Santo Tomás, Los Ángeles 4441171, Chile; danielreyes@udec.cl (D.R.-M.); kkracht@santotomas.cl (K.K.-S.); 3 Doctorado en Psicología, Facultad de Ciencias Sociales, Universidad de Concepción, Concepción 4070409, Chile; 4 Escuela de Kinesiología, Facultad de Ciencias de la Salud, Universidad Católica Silva Henríquez, Santiago 8330225, Chile; icigarroac@ucsh.cl; 5 CIAM Physical Literacy Research Centre, Faculty of Education, Social Science and Humanities, Universidad de La Frontera, Temuco 4811230, Chile; jaime.carcamo@ufrontera.cl; 6 Department of Physical Education, Universidad de La Frontera, Temuco 4811230, Chile; nmartinez@usch.cl; 7 Programa de Doctorado en Didácticas Especificas, Didáctica de la Educación Física, University of Valencia, 46010 Valencia, Spain; 8 ImFINE Research Group, Department of Health and Human Performance, Universidad Politécnica de Madrid, 28040 Madrid, Spain; margarita.perez@upm.es; 9 GALENO Research Group, Department of Physical Education, Faculty of Education Sciences, University of Cadiz, 11510 Puerto Real, Spain; alberto.grao@uca.es; 10 Instituto de Investigación e Innovación Biomédica de Cádiz (INiBICA), 11009 Cadiz, Spain; 11 Research Centre in Physical Activity, Health and Leisure (CIAFEL), Faculty of Sport, University of Porto (FADE-UP), 4050-313 Porto, Portugal; jmota@fade.up.pt; 12 Faculty of Educational Sciences, University of Jaén, 23071 Jaén, Spain; arariza@ujaen.es; 13 Laboratorio de Actividad Física, Salud y Rendimiento Humano, Departamento de Kinesiología, Universidad Metropolitana de Ciencias de la Educación, Santiago 7780450, Chile; fernando.munoz_h@umce.cl; 14 Department of Physiotherapy, Faculty of Medicine, Health, and Sports, European University of Madrid, 28670 Madrid, Spain; 15 BHF Glasgow Cardiovascular Research Centre, Institute of Cardiovascular and Medical Science, University of Glasgow, Glasgow G12 8TA, UK; carlos.celis@glasgow.ac.uk; 16 Human Performance Lab, Education, Physical Activity, and Health Research Unit, Universidad Católica del Maule, Talca 3480112, Chile; 17 High-Altitude Medicine Research Centre (CEIMA), Universidad Arturo Prat, Iquique 1110939, Chile

**Keywords:** physical activity, motor skills, special education, public schools

## Abstract

Background: The development of motor competencies in childhood can enhance the trajectory of physical activity throughout life. However, few studies have examined the effects of physical activity programs on motor competencies in schoolchildren with special educational needs. Aim: Our aim was to analyze the effects of a video-guided active break program on motor competencies in schoolchildren aged 6 to 10 years with special educational needs. Methods: A prespecified subanalysis of a multicenter randomized controlled trial was conducted with a sample of 161 schoolchildren (7.8 ± 1.1 years, 32% girls) with special educational needs from five public schools in Chile. Participants were assigned to a control group (CG, *n* = 85) with no active breaks or an experimental group (EG, *n* = 76) with active breaks. A 12-week video-guided active break program was implemented in the classroom twice daily, five days per week. The intervention was delivered via a web-based platform. Basic motor competencies were assessed using the MOBAK 1–2 and MOBAK 3–4 tests. Results: A significant time × group interaction was found for object control, *F*(1154) = 11.365, *p* < 0.001, η^2^_p_ = 0.011; jumping, *F*(1154) = 11.047, *p* = 0.001, η^2^_p_ = 0.067; and running, *F*(1154) = 4.881, *p* = 0.029, η^2^_p_ = 0.031. These results indicate that the experimental group showed significantly greater improvements in object control, jumping, and running abilities compared to the control group. Conclusions: The active break program significantly improved motor skills in schoolchildren with special educational needs. The program proved to be both feasible and effective in enhancing students’ motor competencies. School-based guided active break programs may play a role in promoting motor competencies among schoolchildren with special educational needs. Clinical Trial ID NCT06423404

## 1. Introduction

Motor competence is described as the ability to perform goal-directed movements, encompassing both physical and perceptual–motor skills in fine and gross motor activities of daily life [1,2]. Motor competencies are fundamental to holistic childhood development. In this regard, it has been reported that motor competence contributes to children’s physical, mental, and social development [3,4]. In an educational setting, schoolchildren with good motor competencies exhibit better physical health, academic performance, self-esteem, and higher levels of physical activity [1,5]. Similarly, in children with disabilities, motor competence supports holistic development by increasing opportunities and facilitators for social inclusion [6,7].

The school environment has been identified as one of the most effective contexts for promoting motor competence, as it reaches a large number of students [8,9]. Beyond its wide reach, schools also provide teachers and other professionals with access to equipment and facilities for promoting physical activity [10]. Moreover, a systematic review suggests that integrating physical activity into the school day is a key aspect of promoting comprehensive physical activity programs and may enhance motor experiences [11].

Various strategies have been explored to develop motor competencies and increase physical activity in schoolchildren. While evidence exists on the effects of different physical activity interventions on children’s and adolescents’ motor competence, there remains a critical gap in research specifically within school settings, mainly focusing on students with special educational needs (SEN). This gap is particularly significant because school-aged children with SEN constitute a vulnerable group that has received less support for engaging in physical activity [12].

In Chile, this concern is underscored by national data: the first study on physical activity habits among people with disabilities [13] indicate that 83% of participants aged 13 to 17 fall within the inactive range according to World Health Organization (WHO) standards. Additionally, in the first national report on physical activity among children and adolescents with disabilities, Chile received low scores for most indicators [14]. Such data highlight a notable participation gap when comparing children with and without disabilities, which places the former group at a distinct disadvantage.

Despite some public policy efforts over the last decade, these have not been reflected in increased physical activity or improved related behaviors among schoolchildren with intellectual disabilities [15]. Taken together, these findings underscore the urgent need for substantial changes and the adoption of a comprehensive approach to effectively address this crisis [14].

Consistent with this perspective, García-Hermoso (2024) emphasizes the importance of physical activity for schoolchildren with disabilities and argues that promoting their participation requires a careful consideration of both facilitators and barriers [16]. Addressing these factors is crucial, as removing obstacles will enable children with disabilities to access the benefits of physical activity across various school environments, including classroom-based activities [17].

A particularly promising tool for developing motor competencies in the school context is the implementation of Active Breaks (ABs). These consist of short periods of physical activity integrated into the school schedule, increasing daily physical activity levels without reducing instructional time [18]. Beyond merely increasing physical activity, ABs have been shown to improve concentration, task organization, and working memory [18,19]. Consequently, ABs have demonstrated effectiveness in enhancing academic outcomes, making them an attractive strategy for teachers and schools [20].

However, there is limited evidence on the effects of ABs in schoolchildren with disabilities. Some studies suggest that classroom-based ABs can increase physical activity levels and reduce sedentary behavior in children with intellectual disabilities, while also potentially benefiting their working memory [21,22,23]. Nevertheless, further research is needed to determine whether this strategy provides additional benefits for this population, particularly in terms of motor competencies, given their importance for holistic development [24]. The present study aimed to examine the effects of a video-guided AB program with curriculum-based content implemented in the classroom on motor competencies in primary schoolchildren with SEN from public schools in the Biobío region of Chile. Analyses were adjusted for school, sex, age, and participation in extracurricular activities, as previous research has shown that these factors significantly influence the development of motor competencies in school-aged children [1,4,5,10].

We hypothesize that the video-guided AB program will lead to significant improvements in motor competencies among children with SEN compared to baseline measures. This study uniquely contributes to the field by [18,19] focusing specifically on classroom-based interventions tailored to students with SEN in a Latin American context, an area that remains underexplored in current literature. By integrating curriculum-based content with innovative video guidance, the program offers a scalable and inclusive strategy to promote motor development within the regular school setting.

## 2. Materials and Methods

### 2.1. Study Design

**Figure 1:**
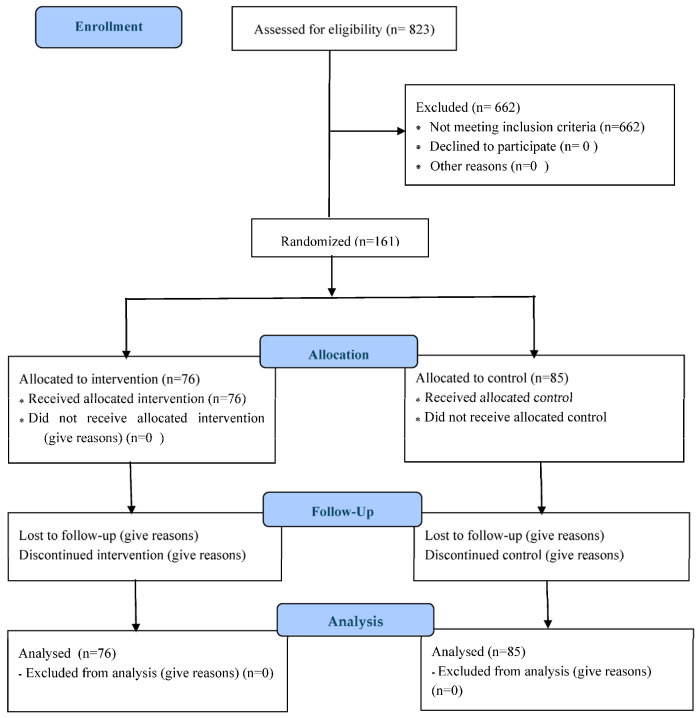
CONSORT 2010 Flow diagram participants.

This study is a pre-specified sub-analysis outlined in the study protocol, focusing on schoolchildren with SEN who were included in the multicenter randomized controlled trial and is registered on ClinicalTrials.gov (NCT06423404), being part of the Active Classes Chile project, conducted between March 2022 and October 2024. The study adhered to the CONSORT (Figure 1) guidelines for clinical trials [25], which provide reporting standards for social and psychological interventions [26]. The study was conducted according the Helsinki’s Ethics Declaration, and all the participants signed a written informed consent before enrolling in the study.

### 2.2. Criteria for School Selection

The selection process for the schools that participated in the experimental evaluation was guided by specific criteria: urban setting, administrative structure, student enrollment, school integration program, and academic performance. Based on data from the educational system, eligible schools were identified in the Biobío region, which has 1022 urban schools. Rural schools were excluded due to limitations such as smaller sample sizes and the presence of multigrade classrooms. The focus was exclusively on public schools, categorized as municipal, delegated administration, and Local Public Education Services (PELS), totaling 372 potential schools in the region. After excluding schools that offer only secondary education, the selection was reduced to 192 schools. Additionally, non-coeducational schools (two in Arauco and two in Concepción) were excluded.

A crucial criterion was enrollment size, particularly medium-sized schools with between 300 and 500 primary students (both with and without special educational needs), ensuring at least two classes per grade from 1st to 4th grade. This range guarantees a sufficient sample size, excluding schools with fewer than 300 students due to the lack of two classes per grade and those with more than 500 students to avoid the complexities of larger school dynamics, which are assumed to have a higher likelihood of conflicts in the school climate. Consequently, the selection was reduced to 41 schools.

Academic trajectory, according to the classification of the Agency for Educational Quality (ACE), is categorized as insufficient, low–medium, medium, and high. This study focused on schools classified as “medium,” whose performance aligns with academic and personal/social development expectations relative to their socio-demographic context. Based on these criteria, the group of eligible schools was reduced to 23: 5 in Arauco, 6 in Biobío, and 12 in Concepción.

For the final selection, five schools were chosen (two in the provinces of Concepción and Biobío and one in the province of Arauco) with a SIP, as it ensures a sample of at least seven students with SEN per class. In addition, the enthusiasm and commitment of the educational leadership, staff, and teachers, as well as the school’s connectivity and resources, were considered.

### 2.3. Participants

The estimated target population consisted of 1080 students, both with and without Special Educational Needs (SEN), from five public schools in the Biobío region of Chile. The final sample included 161 students aged 6 to 10 years with SEN, enrolled in the School Integration Program (SIP). This program provides guidelines to foster the inclusion of all students, aligning with global initiatives that promote diversity and inclusion at all levels of the education system [27]. Additionally, the SIP includes the participation of students with both temporary and permanent needs.

Temporary Special Educational Needs (TSEN) refer to those requiring additional support and resources to access or progress through the curriculum for a specific period of their schooling [28]. In contrast, Permanent Special Educational Needs (PSEN) are learning and participation barriers that certain students experience throughout their education due to a disability [28].

In this study, students with TSEN included those diagnosed with attention deficit disorder (12%), specific learning difficulties (12%), borderline intellectual functioning (12%), and language disorders (7%). Regarding PSEN, participating students had diagnoses of autism spectrum disorder (31%), intellectual disability (24%), and hearing impairment (2%).

The sample size for this pre-specified sub-analysis was determined by the total number of schoolchildren with SEN included in the multicenter randomized controlled trial (161 out of 823 participants). This figure represents 100% of the identified and eligible SEN population from the main study, thereby ensuring representativeness. The sample size is consistent with similar studies and is considered sufficient to detect moderate effects and support the internal validity of the findings for this subgroup.

### 2.4. Curriculum-Based Active Break (AB) Intervention Program

#### 2.4.1. Program Development

The AB program was designed and developed based on a systematic review previously conducted by the research team [29]. Based on the findings of this review, the optimal type and duration of the intervention, the frequency and intensity of the most effective exercises, and the video-guided modalities with curricular content for the active breaks were determined.

#### 2.4.2. Development of an Interactive Web Platform for AB

An interactive web platform for AB, named Active Classes, was created. The platform was designed and developed using modern technologies and frameworks to ensure an optimal user experience, ease of maintenance, and scalability.

The platform features three access profiles: (i) Administrator Profile—Used by the research team for overall platform management, including registering, creating, and removing educational centers, monitoring school usage, creating challenges, and analyzing data. (ii) School Climate Coordinator Profile—Managed by a professional at each school, this profile allows for the registration and removal of teachers within the institution and the monitoring of challenges and progress at different grade levels. (iii) Teacher Profile—Enables teachers to create and implement AB in the classroom and track the progress of their challenges.

The platform provides a repository of exercises used in the AB program, consisting of coordination exercises (bilateral body coordination) and fundamental motor skills (balance, jumping, locomotion), previously validated. Similar programs exist in Europe [30].

These exercises are demonstrated on the platform by two animated characters designed specifically for this project, promoting student engagement and adherence. The exercises are performed in pairs to encourage cooperative learning. The platform included coordination exercises such as lateral jumps, jump rope, forward and backward jumps, skipping, jumping jacks, and muscle strength exercises such as squats.

Additionally, Active Classes incorporates a gender perspective, acknowledging that boys and girls have equal learning potential and technological development opportunities. The platform also fosters cooperative learning spaces through interactive exercises and classroom inclusion, allowing students to participate with animated characters that verbalize questions and response options. Furthermore, the platform includes an educational resource section with adapted exercises for students with reduced mobility. While Active Classes offers an initial set of AB exercises, each teacher has a personalized account, allowing them to apply and design their own AB sessions. To ensure successful implementation of the active breaks, teachers received ongoing support and supervision throughout the 12-week intervention period.

#### 2.4.3. Active Classes Program and Active Break Specifications

The Active Classes program was designed for students in 1st through 4th grade. Each active break lasts between 5 to 10 min and is performed at moderate to vigorous intensity. The breaks are implemented by classroom teachers in alignment with the academic content being taught (language, mathematics, natural sciences, social sciences, and English), five days a week, twice per day, over a 12-week period. The scheduling of AB sessions was adapted to accommodate school timetables, ensuring they are integrated as an interruption within corecurricular subjects—rather than at the beginning or end of a class.

**Figure 2:**
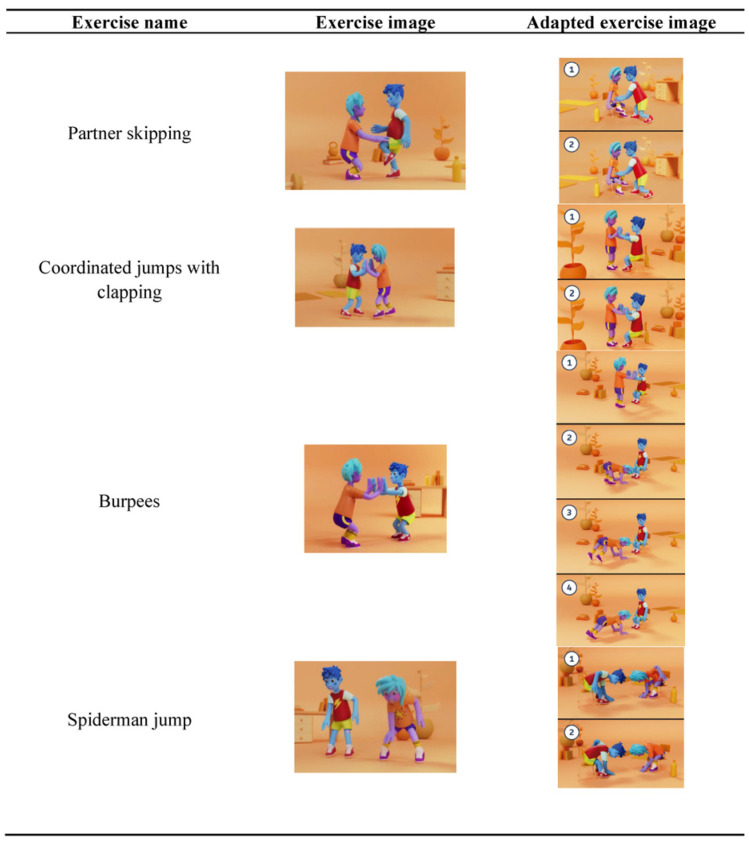
Adapted exercices in the “Active Classes” platform.

Each active break consists of three phases: (1) Welcome and Preparation, (2) Core Content, (3) Closure and Cool-down. The core content phase includes adapted exercises to accommodate students with limited mobility (Figure 2).

### 2.5. Variables and Instruments

#### 2.5.1. Basic Motor Competences

Motor competence was assessed using the MOBAK test battery [31,32] which is widely used across multiple countries due to its high reproducibility. This battery evaluates the status and development of basic motor competencies during the early years of schooling, with task difficulty adjusted according to students’ grade levels. The MOBAK 1–2 test is applied to students in 1st and 2nd grade, while the MOBAK 3–4 test is used for 3rd- and 4th-grade students. Both tests assess motor competence in two dimensions: “Object Control” (comprising tasks such as throwing, catching, dribbling with the hand, and dribbling with the foot) and “Self- movement” (including jumping, running, balancing, and rolling). These tasks increase in complexity according to the students’ grade level. For this study, the MOBAK 1–2 and MOBAK 3–4 tests, previously validated for the Chilean school population [33,34,35], were used. Specifically, the four tasks in the “Object Control” dimension were included, along with the jumping and running tasks from the “Self- movement” dimension. This adaptation was made based on recommendations from professionals working with students with special educational needs [36]. Thus, the scoring scale for “Object Control” ranged from 0 to 8 points (maximum score = 4 tasks × 2 points each), while the “Self- movement” scale ranged from 0 to 4 points (maximum score = 2 tasks × 2 points each). All tests were conducted in participating schools during scheduled physical education classes. The tests were administered in small groups of six participants per test station. To mitigate fatigue and potential measurement errors related to test sequencing, all participants were given a rest period of 3 to 5 min between tests. This variable was assessed by previously trained evaluators.

#### 2.5.2. Covariates

The most common covariates were considered in the analysis of the development of motor competence [37,38]. The covariates analyzed in this study were determined using self-reported questionnaires from the Active Classes Chile project [39]. Sociodemographic variates included sex (boys, girls), age, residential area (urban, rural), distance from school (1–5 blocks, 6–10 blocks, 11–15 blocks, and more than 15 blocks), and access to a park, sports court, or gym near home (no access, with access). School-related variates included school schedule (morning, afternoon, full-day) and school uniform type (sportswear, traditional, mixed). Family-related variates included parental education level (none, primary education, secondary education, and higher education).

### 2.6. Statistical Analysis

The initial characteristics of the participants were described using means and standard deviations (SD) for continuous variables, and frequencies and percentages (%) for categorical variables. Preliminary tests for normality and homogeneity were conducted to assess the sample characteristics. The normality test verified the normal distribution, while the homogeneity test ensured similar variances, thus ensuring valid comparisons between groups. A repeated-measures ANOVA was conducted with Time (before and after the intervention), Group (experimental vs. control), and their interaction (Time × Group). The analysis was conducted independently for each of the eight variables of interest: object movement, throwing, catching, dribbling, bouncing, self-movement, jumping, and running. A Bonferroni correction was applied across the set of eight interaction tests. School, sex, age, and participation in team sports were included in the ANCOVA as covariates. The effect sizes, partial eta squared (η^2^_p_), were calculated for the tests to indicate the magnitude of the effect. The magnitude was interpreted as small at a 0.02 cutoff, medium at a 0.13 cutoff, and large at a 0.26 cutoff [40]. A significance level of *p* < 0.05 was set for all analyses. All analyses were conducted using JASP 0.19.3 statistical software.

## 3. Results

Table 1 reports the main sociodemographic, school-related, family, and physical activity characteristics of the students. A total of 161 students (7.8 ± 1.1 years, 32.3% girls) with special educational needs from five public schools in the Biobío region, Chile, participated in the intervention (Experimental group: *n* = 76, 47.3%; Control group: *n* = 85, 52.7%). With a confidence level of 95%, a heterogeneity of 50%, and a margin of error of 5%. Considering data from previous studies and accounting for an expected dropout rate of 20%, a total of 823 participants were initially recruited from 39 classrooms (two per grade level), each with a maximum of 45 students, including an average of 5 to 6 students with SEN per classroom. The majority of students in both groups resided in urban areas (88.1%) and did not participate in extracurricular workshops (72.1%).

A repeated-measures ANOVA was conducted to evaluate the effect of the video-guided AB program on motor competencies related to object movement (throwing, catching, dribbling, and bouncing) and self-movement (jumping and running) in the Experimental and Control Groups (Table 2). Significant main effects of time were found for all motor competencies, as well as significant time × group interactions, with effect sizes ranging from 0.076 to 0.024. This indicates that, in addition to the general effect of time, significant differences were observed in pre- and post-intervention changes between groups. While the Experimental Group showed an increase in motor competencies, the Control Group exhibited minimal changes.

However, it is important to note that after adjusting for covariates such as school, sex, age, and participation in extracurricular activities, the improvement in jumping was not statistically significant. This suggests that while the program effectively enhanced most motor competencies, certain skills like jumping may require more targeted intervention strategies.

**Figure 3:**
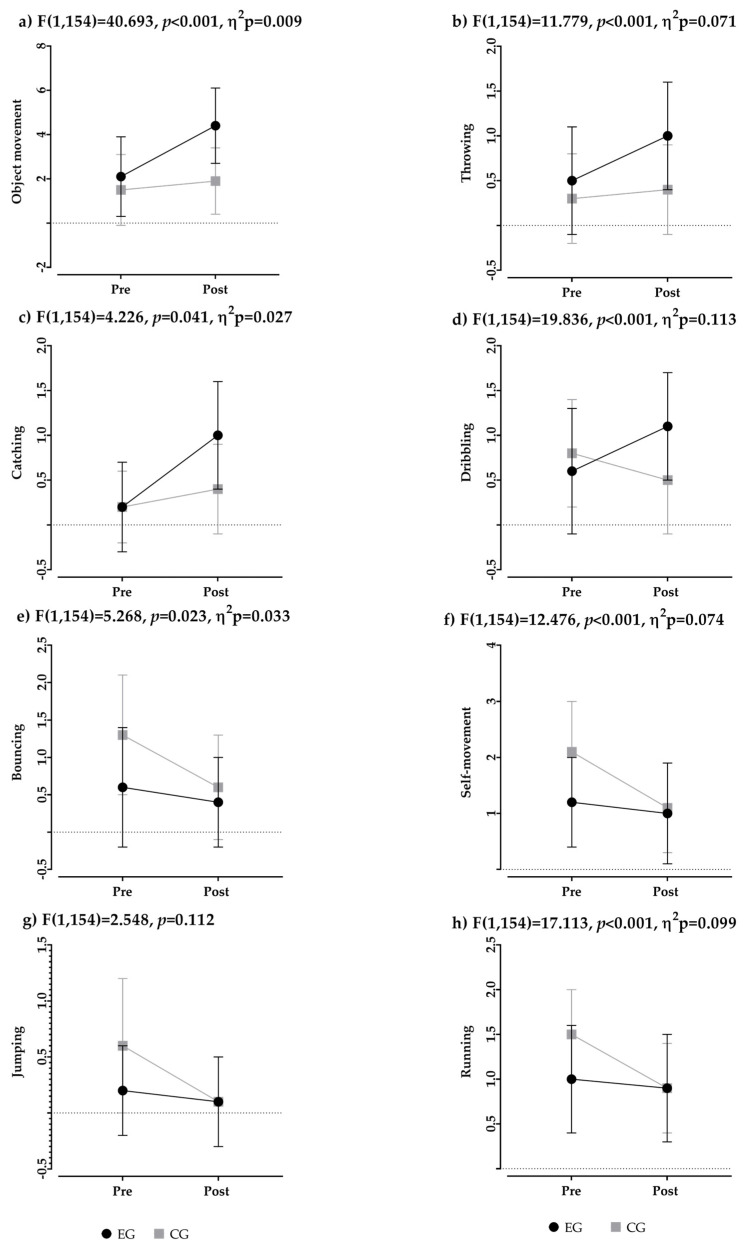
Results of the ANCOVA analysis (pretest vs. post) of the intervention program in the Experimental and Control Groups. A significant pre-intervention difference was observed between the experimental and control groups in object control, *t*(154) = 2.677, *p* = 0.024; rope jumping, *t*(154) = 3.016, *p* = 0.014; and running, *t*(154) = 4.732, *p* < 0.001.

A repeated-measures ANCOVA was conducted, adjusting for covariates (school, sex, age, and participation in extracurricular workshops), to assess the effect of the video-guided AB program on motor competencies (object movement: throwing, catching, dribbling, and bouncing; and self-movement: jumping and running) in the Experimental and Control Groups (Figure 3). After controlling for covariates, a significant main effect of time was observed only for catching (F(1154) = 6.288, *p*-value = 0.013, sample size = 0.039). Conversely, the time × group interaction remained significant for all motor competencies except for jumping (F(1154) = 2.548, *p*-value = 0.112). This suggests that, regardless of the general effect of time, the significant differences in the direction of change between pre- and post-intervention observed in the ANOVA analysis persisted. These findings indicate that, after adjusting for covariates, the differences in motor competencies can be attributed to the specific effect of the video-guided AB program, except for jumping.

## 4. Discussion

### 4.1. Interpretation of Findings

This study examined the effects of a video-guided AB program on motor competence in school-aged children (6 to 10 years) with SEN. The main findings reveal significant improvements in the motor competence of the experimental group compared to the control group. These results align with those reported by Neira-Navarrete et al. (2024), who also observed relevant gains in basic motor skills—particularly body control and object control—following the systematic implementation of physical activity interventions [41]. The findings of this study are particularly relevant given the limited available evidence on active break-based interventions in populations with SEN. Despite the recognized need for further research in this area, the literature identifies only a small number of studies that have systematically evaluated the effects of ABs in schoolchildren with disabilities. For example, Mazzoli et al. [24] and Mero Piedra et al. [42] highlight that while ABs can increase physical activity levels and reduce sedentary behavior in children with intellectual disabilities, their impact on specific motor aspects still requires further exploration [24,42]. Within this context, the present study contributes new evidence by demonstrating the positive impact of ABs on motor competence in children with SEN [24,42].

### 4.2. Comparison with the Literature

These results support the hypothesis that structured active breaks, when tailored to the individual characteristics of students, constitute an effective pedagogical strategy to foster motor development, even in special education settings. The use of audiovisual supports such as videos not only facilitates understanding and execution of the activities but also allows for standardized delivery and sustained engagement, as confirmed in this study and supported by previous research [30,43].

The brief, dynamic, and playful format of ABs appears to be a key factor in their effectiveness, helping to prevent fatigue, ease the transition back to academic tasks, and promote adherence. In this study, high levels of participation and enthusiasm were observed among students, a finding consistent with the experience reported by Sánchez-Matas et al. (2024), who also noted high adherence rates and satisfaction with the intervention [44].

In line with the findings of Ruiz-Ariza et al. (2022), this study provides empirical evidence supporting the use of video-guided ABs as an inclusive, effective, and feasible pedagogical tool for promoting motor competence in students with SEN [45].

The specialized literature consistently shows that regular participation in physical activities in school contexts has significant positive effects on motor development and muscular strength in children with disabilities, Hutzler (2003) and Pérez-Tejero (2014) emphasize that physical activity contributes not only to overall physical fitness but also helps prevent complications associated with sedentary behavior, such as obesity and cardiovascular diseases [46,47]. Moreover, physical activity has been shown to support emotional and social well-being, increasing self-esteem, self-perception, and sense of belonging [48,49].

A systematic review and meta-analysis by Dudley et al. (2022) reinforces these findings, concluding that school-based physical activity promotes holistic student development, with the greatest impact on motor competence, followed by affective, social, and cognitive domains [50]. In this regard, the results of the present study reaffirm the crucial role of physical activity in the integral development of children with disabilities.

Recent studies have also demonstrated that physical play programs can enhance both actual and perceived motor competence. This finding aligns with our results, which showed significant improvements in skills such as throwing, catching, jumping, and running, as assessed using the MOBAK battery [41,51,52]. From a practical perspective, it is important to highlight that the improvement produced by the intervention in the dimension of object control is substantial, since the intervention group showed a mobility from the “need for motor reinforcement” group in the pretest to the “needs improvement” benchmark in the posttest [34].

### 4.3. Contributions to the Literature

The AB program used in this study was designed based on empirical evidence. For instance, jumping exercises have proven effective in improving coordination, balance, speed, and muscular strength [53], and their incorporation through activities such as skipping allowed for the development of multiple dimensions of motor competence. Furthermore, the findings of Gabis et al. (2020) and Kurnaz and Altinkök (2023) support the importance of enhancing coordination skills, particularly in children with ASD or SEN, as these skills require high levels of neuromuscular and cognitive integration [54,55].

The covariate analysis revealed that although the skill of jumping did not show a significant improvement in the time × group interaction, other motor skills did, suggesting that the video-guided AB program was effective in promoting motor development, with the exception of jumping. This skill, being more complex, may require more targeted interventions and longer practice time due to the neuromuscular and cognitive demands involved in its execution [56,57].

An additional point of concern is the high level of sedentary behavior observed in this population, a phenomenon also reported by Martins et al. (2021) and confirmed by our data (76.3% in the experimental group and 81.1% in the control group) [58]. This trend may be explained by various structural and attitudinal barriers within school environments, such as lack of support, inadequate infrastructure, or insufficient teacher training [59]. In Chile, this gap persists, with nearly 30% of students with disabilities lacking access to regular physical activity opportunities [7].

### 4.4. Strengths, Limitations, and Practical Implications

A strength of this study is that it highlights the importance of identifying facilitators for physical activity participation among schoolchildren with special educational needs (SEN) within the classroom setting. Another key aspect is the use of a validated assessment battery across multiple countries, allowing for comparative studies and reference analyses. This is particularly relevant given the limited availability of psychomotor evaluation tools specifically adapted for children with disabilities. As a result, many educational institutions rely on reference values designed for neurotypical children, leading to subjective interpretations [60]. Therefore, the findings contribute to addressing this gap in current knowledge by providing reference values for this population while also reporting the effects of an AB intervention program—an aspect that has not been previously documented.

Among the study’s limitations, we acknowledge the need for future AB intervention studies to incorporate device-based measurements of physical activity. Although the sub-analysis included all available schoolchildren with SEN, this may have limited the ability to conduct stratified analyses by diagnostic subgroups. It is essential to recognize that children with SEN may experience physical, sensory, mental, and cognitive disabilities, as well as learning, emotional, and social difficulties. Consequently, these conditions cannot be approached from a homogeneous perspective [61]. Additionally, the lack of significant improvement in jumping performance after adjusting for relevant covariates points to the need for further investigation into tailored intervention components. This suggests that while the program may benefit some motor skills, others like jumping might require more specialized or intensive approaches.

In the case of students with pervasive developmental disorders (PDD), baseline motor limitations may influence motor skill acquisition, in addition to environmental factors. For instance, in Down syndrome, beyond intellectual disability, structural alterations in the central nervous system are present, hypotonia, hyperlaxity, reduced muscle strength, impaired proprioception, and inadequate postural control [62]. Similarly, in autism spectrum disorder (ASD), alterations in the frontal lobe have been associated with executive function deficits and motor planning difficulties. Additionally, approximately 50% of children with ASD exhibit baseline moderate hypotonia alongside sensory processing abnormalities, which contribute to atypical postural patterns, toe-walking, and stereotyped movements [63]. These factors result in baseline deficits in motor skills such as balance, manipulative abilities, agility, speed, flexibility, muscle strength, and aerobic capacity compared to neurotypical children.

For these reasons, we recommend incorporating active breaks into educational systems of different countries, particularly at the primary school level. Given the developmental characteristics of children at this stage, play-based activities support and promote overall well-being. Specifically, we suggest integrating active break initiatives into classroom planning to provide evidence of their benefits for learning outcomes in all students, especially those with SEN. In terms of future directions, we propose a long-term follow-up to evaluate the sustained effects of active breaks over time. Additionally, future studies should include additional variables that further emphasize the importance of active learning strategies in the classroom and their role in enhancing the educational experiences of students with special educational needs.

## 5. Conclusions

The active break program proved to be both feasible and effective in enhancing motor competencies among schoolchildren with special educational needs. These findings suggest that implementing guided active break programs within the school environment may play a crucial role in promoting motor development in this population. Furthermore, the results emphasize the importance of integrating movement-based methodological strategies into the school curriculum, fostering both learning and well-being among students with special educational needs. It is a low-cost, inclusive program that has demonstrated positive effects on the learning of schoolchildren with SEN, and its implementation could be transferable to educational systems with contexts similar to those of this study.

## Figures and Tables

**Table 1:** Demographic characteristics of the sample.

Variables	Total (*n* = 161)	Experimental Group (*n* = 76; 47.3%)	Control Group (*n* = 85; 52.7%)
Age, *M* ± *SD*	7.8 ± 1.1	7.9 ± 1.2	7.6 ± 1.0
Gender, *n* (%)			
Boys	109 (67.7)	50 (65.7)	59 (69.5)
Girls	52 (32.3)	26 (34.3)	26 (30.5)
Zone, *n* (%)			
Urban	142 (88.1)	63 (82.8)	79 (92.9)
Rural	19 (18.9)	13 (17.2)	6 (7.1)
School			
1	43 (26.7)	0 (0.0)	43 (50.5)
2	42 (26.2)	0 (0.0)	42 (49.5)
3	20 (12.3)	20 (26.4)	0 (0.0)
4	31 (19.3)	31 (40.8)	0 (0.0)
5	25 (15.5)	25 (32.8)	0 (0.0)
Grade, *n* (%)			
1°	27 (16.8)	13 (17.1)	14 (16.4)
2°	37 (23.0)	16 (21.1)	21 (24.7)
3°	51 (31.7)	21 (27.6)	30 (35.4)
4°	46 (28.5)	26 (34.2)	20 (23.5)
After-school workshop			
Yes	45 (27.9)	24 (31.5)	21 (24.7)
No	116 (72.1)	52 (68.5)	64 (75.2)

*n*: sample size. Mean (*M*) and standard deviation (*SD*) were used for continuous variables. Frequencies (%) were used for categorical variables.

**Table 2:** Results of the ANOVA analysis (pretest vs. post) of the intervention program in the Experimental and Control Groups.

Variables	Group	Pretest	Posttest	Time		Time × Group	
		*M* ± *SD*	*M* ± *SD*	*F (p)*	*η^2^_p_*	*F (p)*	*η^2^_p_*
Object movement	EG	2.1 ± 1.8	4.4 ± 1.7	156.104 (<0.001) ***	0.495	40.693 (<0.001) **	0.204
	CG	1.5 ± 1.6	1.9 ± 1.5				
Throwing	EG	0.5 ± 0.6	1.0 ± 0.6	26.630 (<0.001) ***	0.143	23.301 (<0.001) ***	0.128
	CG	0.3 ± 0.5	0.4 ± 0.5				
Catching	EG	0.2 ± 0.5	0.8 ± 0.6	54.911 (<0.001) ***	0.257	19.860 (<0.001) ***	0.111
	CG	0.2 ± 0.4	0.3 ± 0.5				
Dribbling	EG	0.6 ± 0.7	1.1 ± 0.6	21.523 (<0.001) ***	0.119	17.693 (<0.001) ***	0.100
	CG	0.8 ± 0.6	0.5 ± 0.6				
Bouncing	EG	0.6 ± 0.8	1.3 ± 0.8	49.596 (<0.001) ***	0.238	19.464 (<0.001) ***	0.109
	CG	0.4 ± 0.6	0.6 ± 0.7				
Self-movement	EG	1.2 ± 0.8	2.1 ± 0.9	84.333 (<0.001) ***	0.347	28.503 (<0.001) ***	0.118
	CG	1.0 ± 0.9	1.1 ± 0.8				
Jumping	EG	0.2 ± 0.4	0.6 ± 0.6	38.607 (<0.001) ***	0.195	17.651 (<0.001) ***	0.100
	CG	0.1 ± 0.4	0.1 ± 0.4				
Running	EG	1.0 ± 0.6	1.5 ± 0.5	47.770 (<0.001) ***	0.231	13.071 (<0.001) ***	0.076
	CG	0.9 ± 0.6	0.9 ± 0.5				

EG: Experimental Group; CG: Control Group. *M*: Mean; *SD*: Standard deviation. *** *p*-value < 0.001; ** *p*-value < 0.01.

## Data Availability

The data presented in this study are available on request from the corresponding author due to restrictions imposed to safeguard the identity of the subjects studied and to comply with applicable ethical regulations.
